# Assembling semiconducting molecules by covalent attachment to a lamellar crystalline polymer substrate

**DOI:** 10.3762/bjnano.7.70

**Published:** 2016-06-02

**Authors:** Rainhard Machatschek, Patrick Ortmann, Renate Reiter, Stefan Mecking, Günter Reiter

**Affiliations:** 1 Institute of Physics, University of Freiburg, Hermann-Herder-Strasse 3, 79104 Freiburg, Germany; 2 Chair of Chemical Materials Science, Department of Chemistry, University of Konstanz, Universitätsstrasse 10, 78457 Konstanz, Germany

**Keywords:** monolayer, nanocrystals, precision polyethylene, semiconducting molecules, single crystals

## Abstract

We have investigated the potential of polymers containing precisely spaced side-branches for thin film applications, particularly in the context of organic electronics. Upon crystallization, the side-branches were excluded from the crystalline core of a lamellar crystal. Thus, the surfaces of these crystals were covered by side-branches. By using carboxyl groups as side-branches, which allow for chemical reactions, we could functionalize the crystal with semiconducting molecules. Here, we compare properties of crystals differing in size: small nanocrystals and large single crystals. By assembling nanocrystals on a Langmuir trough, large areas could be covered by monolayers consisting of randomly arranged nanocrystals. Alternatively, we used a method based on local supersaturation to grow large area single crystals of the precisely side-branched polymer from solution. Attachment of the semiconducting molecules to the lamellar surface of large single crystals was possible, however, only after an appropriate annealing procedure. As a function of the duration of the grafting process, the morphology of the resulting layer of semiconducting molecules changed from patchy to compact.

## Introduction

Organic chemistry constantly provides new, high-performance semiconducting molecules for organic electronic applications [1]. However, efforts to develop strategies for assembling these molecules into functional devices are often less sophisticated compared to the concepts applied for their synthesis. As a rapid and facile technique, spin coating is frequently the method of choice to integrate semiconducting molecules into devices. Yet, effects like crystallization or dewetting may lead to films with a complex morphology that differs substantially from the homogeneous, closed and largely defect-free film, which is required for good charge transport. One has to take into account that differences in device morphology directly translate into differences in device performance. Therefore, it may become difficult to distinguish between a molecule, which is poorly conducting and a poorly fabricated device.

While annealing procedures can help to homogenize the morphology of the spin coated films, those procedures have to be adopted and optimized for each individual molecule. Thus, standard annealing procedures have varying success. Here, we demonstrate an approach for the formation of homogeneous, ultrathin semiconducting layers, which is expected to be applicable universally to a broad range of semiconducting molecules: By covalently attaching functional molecules to an insulating, smooth and crystalline substrate, we aim at creating a compact and homogeneous monolayer of semiconducting molecules. This method can be applied to many semiconducting molecules independent of their chemical structure and physical properties, provided that these molecules possess a functional linker for covalent attachment. The challenge of this concept lies in the generation of an appropriate substrate, which is decorated by an ordered array of functional groups. In addition, this substrate should be extremely smooth so that the order of the semiconducting molecules in the monolayer is not perturbed by roughness, i.e., molecules are not separated by ‘hills’ or ‘valleys’ on the substrate.

Promising candidates for such a material are polymers with precisely spaced side-groups and good insolating properties, like for example polyethylene, which form crystals of uniform thickness. Usually, when polymers crystallize, they form thin lamellar layers of ordered but folded chains. The thickness of these lamellae is determined by crystallization kinetics [2–3]. During crystallization of polymers possessing occasional side-groups which cannot be integrated in the crystal lattice, these side-groups are typically excluded from the crystalline lamellae. Thus, for precisely spaced side-groups, the lamellar thickness is basically predetermined by the distance between two consecutive side-groups [4–6]. Chain folding is expected to take place at the position of the side-groups. Consequently, the surface of such lamellar crystals will be covered by side-groups. With an appropriate choice of these groups, chemical reactions may be performed at such surfaces. Thus, such lamellae covered with side-groups represent smooth surfaces with functional groups, which allow for a covalent attachment of semiconducting molecules. Subsequently, the resulting monolayer of semiconducting molecules on the surface of an insulating polymer crystal can be electrically contacted to form the basis of an ideal organic transistor. Such a model system should allow for the measurement and comparison of intrinsic electronic properties of a broad range of semiconducting molecules.

However, the typically rather small size of polymer single crystals represents a drawback of the method described above. This small size complicates the application of electrical contacts to the device. In order to overcome this problem, we pursued an enhanced variant of the aforementioned approach: We attempted to create ordered monolayers of nanocrystals made of a precisely side-branched polymer. Those ordered monolayers of nanocrystals should have surfaces similar to the surface of single crystals, but can be prepared on much larger scales, as has been demonstrated for colloidal particles for which ordered monolayers of square centimeter sizes were achieved [7].

The polymer, which we chose for our experiments, was polyethylene with carboxyl groups at every 45th carbon atom, denoted as CPE45. The carboxyl groups were chosen for two reasons: Firstly, they can be used for the covalent attachment of semiconducting molecules and secondly, they can be negatively charged. Therefore, CPE45 allows for the synthesis of nanocrystals which are electrostatically protected against aggregation in an aqueous dispersion [8]. We now show how smooth CPE45 surfaces can be produced and under which conditions the density of carboxyl groups is sufficient to allow for the formation of a compact layer of covalently attached semiconducting molecules. Finally, we will also examine the morphology and spectroscopic properties of the layer of semiconducting molecules.

## Results

### Nanocrystal analysis by AFM

#### The model of an ideal CPE45 nanocrystal

**Figure 1:**
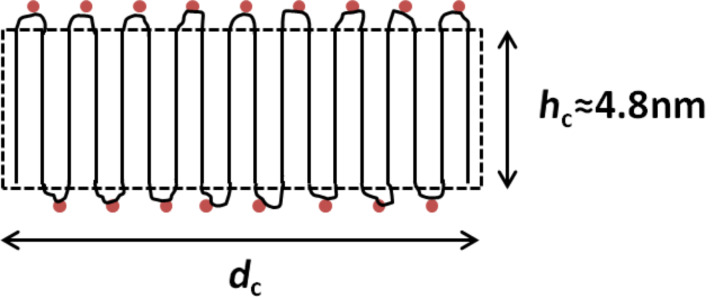
Schematic representation of the cross-section of an ideal CPE45 nanocrystal. The polymer forms sharp, hairpin-like folds at every carboxyl group represented as a red dot.

Nanocrystal dispersions were prepared via nanoprecipitation with the impact of shear forces generated by ultrasonication. By cryo-transmission electron microscopy (TEM) studies on the dispersions, a thickness of the nanocrystals of about 5 nm was found [8]. This observed value matches well with the concept of a polyethylene chain which is regularly folded at every carboxyl side-group. In this consideration, of the 44 methylene units between two consecutive carboxyl groups, about 6 are thought to create sharp, hairpin-like folds [9]. The remaining 38 bonds define the crystal thickness of 4.8 nm, derived from the projected length of 0.127 nm of a carbon–carbon single bond in a crystalline polyethylene lamella (Figure 1) [10].

#### Lateral sizes

**Figure 2:**
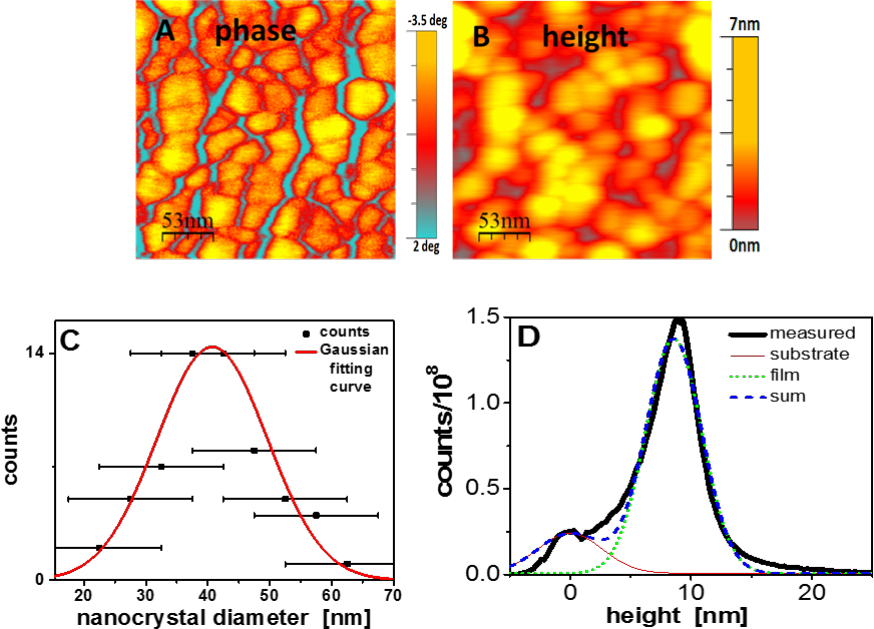
A) Phase image of a close packed region of a monolayer of nanocrystals, obtained by self-assembly on the air–water interface. B) Height image of the same region. C) Statistical analysis of the width of nanocrystals, deduced from a large number of cross-sections taken in the scanning direction, i.e., parallel to the bottom axis. D) Height histogram calculated from a larger area scan of a similar sample as in A) and B), fitted with two Gaussians.

In Figure 2, we show typical phase and height images of a nanocrystal monolayer prepared by assembly of the nanocrystals at the air–water interface. A statistical analysis of the width of individual crystalline objects, deduced from measurements of the cross-section of about 60 different nanocrystals, indicated a characteristic value of the lateral size *d*_c_ of the crystals of about 40 nm and a Gaussian distribution around this value with a full-width-at-half-maximum of about 20 nm. From the Gaussian fitting curve, we deduced a polydispersity of 22%. (Polydispersity is defined as 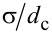, with σ being the standard deviation of 9 nm derived from the Gaussian fitting curve).

Based on the analysis of cryo-TEM images [8], the nanocrystals have a hexagonal, platelet like habit. However, depending on crystallization conditions polyethylene crystals can also exhibit different habits like lozenges or truncated lozenges [10]. The AFM phase image (Figure 2A) suggests nanocrystals slightly varying in size. However, the exact habit cannot be determined due to limited resolution introduced by AFM tips having a radius of curvature of about 10 nm.

**Figure 3:**
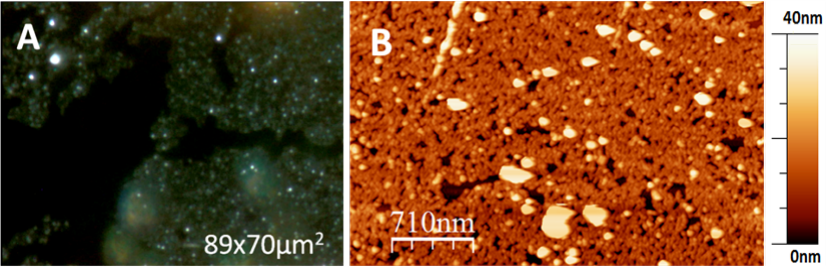
A) Optical dark field image of CPE45 nanocrystal rafts, self-assembled at the air–water interface. B) AFM height image of a raft of self-assembled CPE45 nanocrystals.

The nanocrystals within the monolayer were found to be packed rather densely. However, there were voids between the nanocrystals, as they are hard objects of non-identical size and thus cannot form packings without defects. Furthermore, Figure 2B suggests that not all nanocrystals had the same thickness. We note, however, that the heights measured by AFM are not equal to the thickness of the nanocrystals as the voids between the nanocrystals were partially filled by the amorphous loops and loose chain ends representing the non-crystalline parts of the polyethylene molecules integrated in the nanocrystals. In addition, the spacing between nanocrystals was often smaller than the diameter of the AFM tip, which therefore could not reach the substrate. Accordingly, a smaller value of the crystal height would be detected. Therefore, and within the limitations described above, we have extracted the value for the thickness of the platelet-shaped nanocrystals from large area AFM scans of nanocrystal films, where occasional voids were large enough for the AFM tip to frequently reach the substrate (an example is shown in Figure 3B). The corresponding height histogram (see Figure 2D) showed two peaks: The one centered at 0 nm represents the substrate, while the other peak centered at 8.5 nm represents the most frequently measured thickness of the nanocrystals. The rather unique height value of the film shown in Figure 3B, which consists of some thousand nanocrystals, supports the uniformity of the thickness of the nanocrystals. We note that the value of the nanocrystals thickness clearly exceeded the expected crystal thickness of 4.8 nm. This implies that the overall thickness CPE45 nanocrystals does not depend solely on the thickness of the crystalline core, which is predefined by the distance between regularly spaced side-groups.

### Formation of monolayers of nanocrystals

#### Self-assembly of nanocrystals at the air–water interface

For electrostatically stabilized colloids, self-assembly at the air–water interface has been demonstrated to be an efficient route to generate close packed monolayers with virtually crystalline order up to sizes of square-centimeters [7]. While repulsive forces introduced by surfactants are often used to support the assembly process [7,11], we refrained from adding surfactants as we wanted to avoid to have surfactant molecules included in the monolayers transferred onto a solid substrate. Accordingly, we spread CPE45 nanocrystals from a 1:1 methanol–water dispersion onto a water surface at pH 11. After Langmuir–Schäfer transfer to a silicon substrate, rafts of nanocrystals having sizes up to several hundred square micrometers could be detected via optical microscopy (Figure 3A). AFM images on the micrometer scale show that the rafts consisted indeed of rather close packed nanocrystals (Figure 3B). However, the rafts had many voids and the thicker objects found in the image indicate that the nanocrystals had a certain tendency to stack.

Concerning the origin of voids in the monolayer, one has to keep in mind that the CPE45 nanocrystals vary in size and possibly to some extent in shape. Therefore, nanocrystals could not pack perfectly. Accordingly, CPE45 nanocrystals were not able to form a compact monolayer with a packing density of 1 (i.e., 100% of the area is filled by the nanocrystals). Only a reduced packing density less than 1 was observed. Presently, no theoretical description for the dense packing of objects with hexagonal or lozenge-like particles differing in size is available. Even for particles of a uniform and simple shape like disks or squares, their maximum packing density is affected by their size distribution. For monodisperse disks in a hexagonal two-dimensional arrangement, a maximum packing density of 90.7% can be achieved [12]. For a square packing of monodisperse disks, the packing density is reduced to 79% [13]. For disks having a variation in size similar to the dispersion of CPE45 nanocrystals, no long range order can be achieved and a maximum packing density of about 84% is expected [14].

The area coverage of the nanocrystal monolayer was calculated using Gwyddion software [15]. The AFM image was corrected for the AFM tip shape using a deconvolution algorithm. The tip shape was characterized by parameters provided by the manufacturer. In order to deduce the packing density of a nanocrystal film from an AFM image, one has to identify the reference height level of the substrate, which crucially depends on the definition of a threshold value. Therefore, the height histograms were fitted with two peaks, representing the substrate and the monolayer, respectively. We set the threshold value in a way that 90% of the height values belonging to the substrate (corresponding to the area of the substrate peak) were below the threshold value. Thus, the probability of erroneously considering a point on the substrate as the surface of a nanocrystal was below 10%, when defining the threshold value in this way. We used the same threshold value on three independent images of the same film and found an average area coverage of 78 ± 4%, i.e., a packing density of 0.78 ± 0.04.

#### Compression of layers of nanocrystals on a Langmuir trough

As shown above, when deposited on the air–water interface, the nanocrystals assembled to isolated rafts (or islands). In order to bring these rafts closer, with the aim to form a large compact film, we deposited the nanocrystals onto the air–water interface of a Langmuir trough, where we could compress the deposited layer by moving the barriers of the Langmuir trough. We found that the recorded surface pressure depended on the compression rate: When we compressed films of CPE45 nanocrystals at relatively high compression speeds (ca. 1 mm/min), we recorded surface pressures increasing to values as high as Π = 10 mN/m, indicating repulsive interactions between the nanocrystals. However, when the films were compressed at much lower speeds (≈0.05 mm/min), we recorded only minor increases of the surface pressure (up to Π ≈ 0.5 N/m). Moreover, when allowing for relaxations (i.e., measuring the surface pressure as a function of time without moving the barriers), the surface pressure of films compressed at high rates decayed rapidly. We therefore concluded that rapidly compressed layers of nanocrystals exhibited a relaxation or aging process due to various rearrangement processes of the nanocrystals, expressed, e.g., by a rather fast rearrangement step which might be related to the repositioning of rafts of nanocrystals and a rather slow rearrangement step, possibly related to the repositioning of nanocrystals within individual rafts [16]. Accordingly, the pressure decay could be described reasonably well by the sum of two exponential decays. (Surface pressure diagrams of CPE45 nanocrystal films recorded during compression and relaxation are shown in Supporting Information File 1)

**Figure 4:**
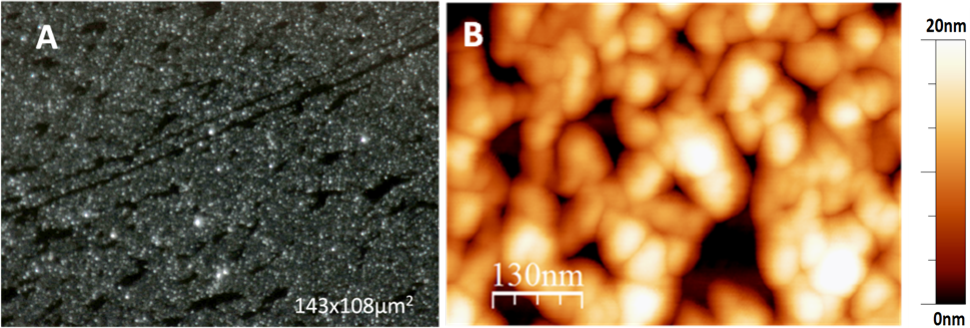
A) Optical dark field image of a transferred film of nanocrystals compressed on the Langmuir trough. B) AFM image of another film showing that such compression on the Langmuir trough led to a packing of the nanocrystals similar to their arrangement in the rafts of nanocrystals resulting from self-assembly at the air–water interface.

From AFM images, we find that packing and local order in compressed films (see Figure 4B) was not significantly different from films of nanocrystals self-assembled at the water–air interface without being compressed by moving barriers (see Figure 3B). The packing of nanocrystals was clearly not generating long-range order, most likely because nanocrystals were varying in size and possibly in shape. When exceeding a certain packing density, particles jam [17], which may impede rearrangements. Further compression may not be possible without expelling of nanocrystals from the monolayer [18] or without buckling of the film [19].

#### Chemical attachment of semiconducting molecules to the surfaces of CPE45 nanocrystals

We have produced rather close packed monolayers of CPE45 nanocrystals with packing densities of about 0.78, having a size of several thousand square micrometers. While the application of electrical contacts to these layers may be difficult, these layers of nanocrystals are rather smooth and covered with functional groups allowing for covalent attachment of molecules. Thus, we could functionalize these layers of nanocrystals by chemically grafting semiconducting molecules that interact via π-orbitals. For a densely grafted layer of such molecules, potentially significant overlap between their π-orbitals can be achieved, resulting in efficient charge carrier transport [20] parallel to the nanocrystal surface.

For a proof of principle, an amino functionalized perylene 3,4-dicarboximide derivative (**1**) was chosen. We synthesized molecules **1** according to the procedure given in [21]. For an isolated molecule **1**, fluorescence emission is quenched due to the presence of the electron donating amine group [22]. Upon chemical reaction with a carboxyl group, the electron donating character is reduced and the molecule becomes fluorescent. Therefore, the chemical attachment of **1** to the nanocrystal surface can be demonstrated directly via the observation of fluorescence emission. In addition, upon π–π stacking of molecule **1**, fluorescence emission exhibits large spectral shifts [23]. Therefore, we can draw conclusions about the packing density of semiconducting molecule **1** attached to CPE45 nanocrystal surfaces without having to resort to conductivity measurements. As a reference surface, which certainly was densely covered by carboxyl groups, and for comparison, we have used a film of spin-coated polyacrylic acid to which we attached **1** in an analogous way as to the CPE45 nanocrystals. Both films were kept for one week in a solution containing a large excess of **1** and the coupling reagents, resulting in functionalization of the surface via chemically grafting molecules **1** to the carboxyl groups present at the surface.

**Figure 5:**
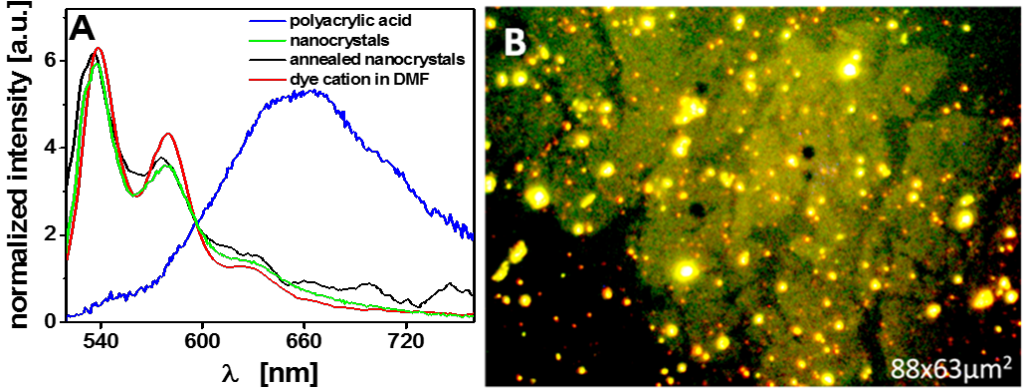
A) Emission spectra of nanocrystals with chemically attached **1**, the protonated perylene dye in solution and the polyacrylic acid film with chemically attached perylene dye. The spectra from the as deposited and annealed nanocrystals were recorded from a drop-casted film. B) Fluorescence image of a monolayer raft of nanocrystals after reacting this layer for one week with a solution of **1**. Red and green spots are probably the guanidinium side-product of the coupling reaction [24], as these emissions were absent before functionalization.

Based on the observed low fluorescence intensity, we concluded that the number of perylene molecules attached to CPE45 nanocrystal surfaces was low, suggesting that the chemical reaction was not very successful. The observed emission spectrum from a layer of nanocrystals covered with **1** was almost identical to the emission spectrum of protonated and therefore non-aggregated **1** in a dilute DMF solution (red and green curve in Figure 5A). On the other hand, the functionalized polyacrylic acid film exhibited a red shifted, broadened emission spectrum (blue curve in Figure 5A), indicating dense packing of perylene molecules [25]. Thus, the low density of perylene molecules on the surfaces of CPE45 nanocrystals was caused by a low density of accessible carboxyl groups and not by a low yield of the chemical reaction. In order to allow for the reordering of the fold surfaces of the CPE45 nanocrystals, the films were annealed at 85 °C (which is close to the bulk polymer melting point) for 24 hours. To prevent chains, which have eventually melted during annealing, from adsorbing on top of the nanocrystal surfaces, the still hot samples were immersed in tetrahydrofuran (THF) (which is a rather good solvent for CPE45) at room temperature. We note that CPE45 nanocrystals are stable in THF and do not dissolve at temperatures below 50 °C. However, this annealing and washing procedure did not lead to changes of the emission spectra after reacting the nanocrystals with **1** for several days (black curve in Figure 5A). Thus, annealing of CPE45 did not increase the density of accessible carboxyl groups on the surfaces of the nanocrystals. Similarly, from AFM images, we found no indication for the presence of a layer of chemically attached perylene molecules after functionalization. The length of a perylene molecule **1** is about 1.5 nm. Thus, a densely packed layer of perylene molecules standing upright on the crystal surface could be identified in AFM height images, as will be shown in the following section.

#### Growth of large CPE45 single crystals

Taking into account that due to variations in size and possibly shape of the nanocrystals, rafts of CPE45 nanocrystals contain voids and considering that carboxyl groups on surfaces of CPE45 nanocrystals are only partially accessible for a chemical reaction, we investigated another pathway to produce smooth substrates for covalent attachment of semiconducting molecules. To this end, we prepared large CPE45 single crystals from solution. As single crystals of polyethylene prepared in solution by a self-seeding technique are typically rather small [26] and melt crystallization of polyethylene is highly challenging [27], we employed an alternative method for growing large polymer single crystals, taking advantage of the dependence of the nucleation probability on local super-saturation.

In order to grow defect-free and large single crystals, both nucleation rate [28] and crystal growth rate [29] need to be low. The bulk CPE45 polymer material was dissolved in THF to yield a homogeneous solution with an initial concentration between 0.01 and 0.001 mg/mL. The homogeneous solution was kept in an open vial at an elevated temperature. A silicon substrate was immersed half-way in the solution. Continuous evaporation of the solvent increased the polymer concentration, leading eventually to local super-saturation, i.e., locally the concentration was above the solubility limit. At the three phase contact line, i.e., at the line where substrate, solution and air met, the concentration was highest. Thus, the probability for crystal nucleation was highest at this line. In addition, due to continuous solvent evaporation and the thereby induced convection in the region of the wedge close to the contact line, a constant supply of polymers was flowing towards the contact line and provided the molecules needed for growing crystals. The mechanism which led to local supersaturation and crystallization is rather similar to convective assembly techniques which are frequently applied to create ordered mono- or multilayers of colloidal particles [30].

**Figure 6:**
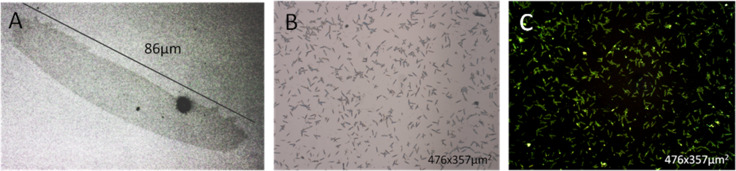
A) Bright field optical micrograph of a single crystalline lamella of CPE45. B) Bright field optical micrograph of an array of multi lamellar crystals of CPE45 with attached **1**. C) Fluorescence micrograph of the same sample as in B.

Temperature, evaporation rate and polymer concentration affect nucleation probability and crystal growth rate. However, pinning of the contact line had the strongest influence on number density and size of the crystals. Therefore, on most samples, we observed arrays of many crystals which all had a length of about 10 micrometers and often consisted of a stack of several lamellae (Figure 6B,C). In a few regions, however, isolated and significantly larger mono-lamellar single crystals were observed (Figure 6A).

Three observations supported our conclusion that we have generated indeed single-crystals: Firstly, the characteristic sheet-like geometry, i.e., a thickness in the nanometer regime paired with a lateral size in the micrometer regime, is typical of polymer crystals [10]. Secondly, their lateral habit is in accordance with the habit of polyethylene single crystals grown at relatively high temperatures [27,31]. Thirdly, the crystals showed lamellar thickening when annealed at elevated temperatures (an AFM image is shown in Supporting Information File 1), a characteristic behavior of lamellar polymer crystals [10].

#### CPE45 single crystals with chemically attached semiconducting molecules

We have shown that the attachment of semiconducting molecules to nanocrystals did not lead to a high density of grafted molecules. Similarly, when we tried to functionalize the solution-grown CPE45 single crystals with **1**, we had very limited success. Thus, we concluded that not all carboxyl groups were accessible at the surfaces of as-grown CPE45 single crystals. Most of the carboxyl groups must have been covered by an amorphous layer which impeded a chemical reaction with **1**. The presence of an amorphous layer surrounding the crystalline core of the lamellar crystals was confirmed by AFM height measurements. Determined by the distance between the side-groups in the backbone of the polymer, the thickness of the crystalline core is about 5 nm. AFM yielded larger values for the thickness of the crystals.

To increase the accessibility of the carboxyl groups on the surfaces of CPE45 single crystals, we applied the same annealing procedure as for layers of densely packed CPE45 nanocrystals. However, for the large CPE45 single crystals, annealing increased the probability of a successful reaction with **1** drastically. Via reordering processes at the fold surface, an increase in density of accessible carboxyl groups was achieved during thermal annealing, which, in turn, was only possible upon inclusion of a layer of carboxyl groups into the stack of crystalline lamellae. A detailed description of the morphological changes that could be observed during thermal annealing, side-group inclusion and reordering of the fold surface of CPE45 single crystals will be presented in a future publication. Here, we focus mainly on the morphology and the evolution of the layer of semiconducting **1** molecules on top of the crystal surface achieved through the grafting procedure*.*

**Figure 7:**
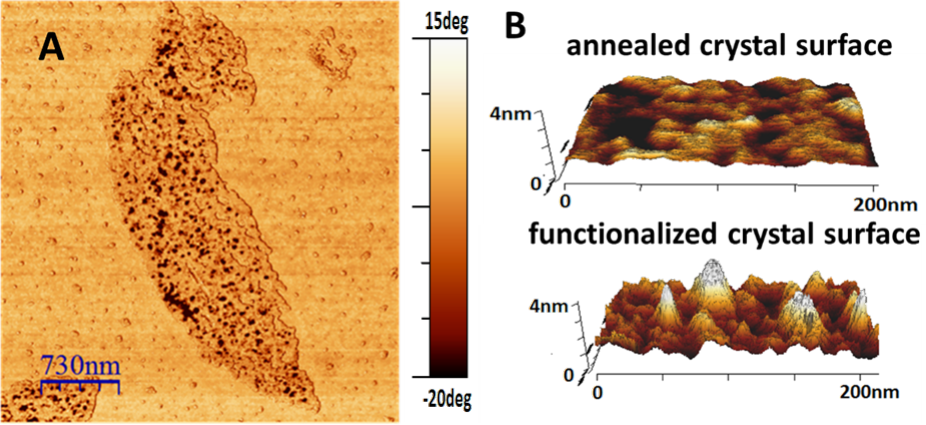
A) AFM phase image of an annealed CPE45 single crystal after functionalization with **1** for one day, showing soft islands growing exclusively on the crystal surface. B) Comparison of the surface roughness of an annealed CPE45 single crystal before functionalization and after functionalization with **1** for one day. The strong increase of surface roughness indicates that semiconducting molecules **1** were attached to the crystal surface.

In AFM images, the surface of the annealed crystal before functionalization with **1** and the silicon substrate next to the crystal showed almost the same phase contrast, indicating that both surfaces exhibited similar viscoelastic properties (Figure 7A). However, after chemically grafting **1**, several ca. 100 nm wide and significantly softer or stickier [32] islands with heights between 1.5 nm and 4 nm were found on top of the crystal (Figure 7B). By contrast, no such islands were detected on the substrate. These islands grew selectively on the crystal surface and therefore were most likely generated by the semiconducting molecules. Their softness can be explained by a rather low number of attached dye molecules per island: When packing was not sufficiently dense, molecules could bend or be pushed sideways under the load applied through the cantilever, allowing the AFM tip to penetrate the semiconducting layer.

Initially, the probability for a chemical reaction of the surface functional groups with **1** was identical throughout the whole crystal surface. Thus, during the early stage of the grafting procedure, semiconducting molecules were distributed homogeneously. However, at later stages, interactions between molecules **1** may have caused a heterogeneous distribution. Perylene molecules have strong π–π interactions [25]. Thus, already attached molecules could attract the incoming molecules via these π–π interactions, and keep them close to the crystal surface for rather long times. Thereby, the probability of these newly arriving molecules to graft chemically to adjacent positions would be enhanced. Such a process could promote the growth of islands. In addition, the detection of islands did not imply that there were no **1** molecules outside of the islands. Their number density may, however, have been below the detection limit of AFM.

**Figure 8:**
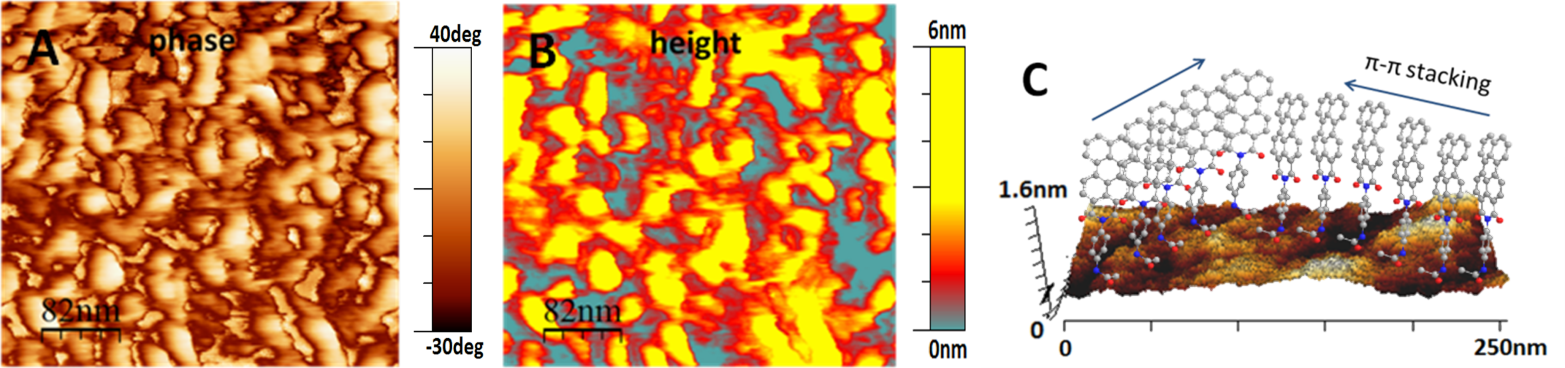
A) AFM phase image of the annealed crystal surface after reacting it for 3 days with a solution of **1**. The difference in phase contrast in the connected domains may be explained by a varying packing density of semiconducting molecules, leading to a more elastic response detected by the AFM tip. B) AFM height image corresponding to the phase image shown in A. C) Schematic representation of the orientation of perylene molecules on the crystal surface. The height values are to scale, but the lateral dimensions of the molecules are strongly exaggerated.

After allowing **1** molecules from a surrounding solution to react with the crystal surface for 3 days, this surface was covered with a kind of network of islands of semiconducting molecules, as shown in Figure 8.

For islands extending 80 nm and more visible in Figure 8B, the highest features were about 5 nm in height. From this observation, we conclude that the molecules did not lie flat on the surface: Typically, for molecules interacting via their planar aromatic π-electron systems, the primary direction of crystal growth is given by the direction in which the π-electron systems stack [33–34]. In our samples, the primary growth direction of the islands was parallel to the surface, indicating that the orbitals of the π-electrons were oriented perpendicular to the surface normal. From the phase image in Figure 8A, we deduced that some parts of the connected structure had viscoelastic properties similar to the hard crystal surface, while other parts were significantly softer. Structures, which appeared elastically in the phase image, were thicker in the height image. This is consistent with a more compact packing of the semiconducting molecules. Accordingly, the AFM tip could not penetrate such domains and they appeared as elastic domains in the phase image.

**Figure 9:**
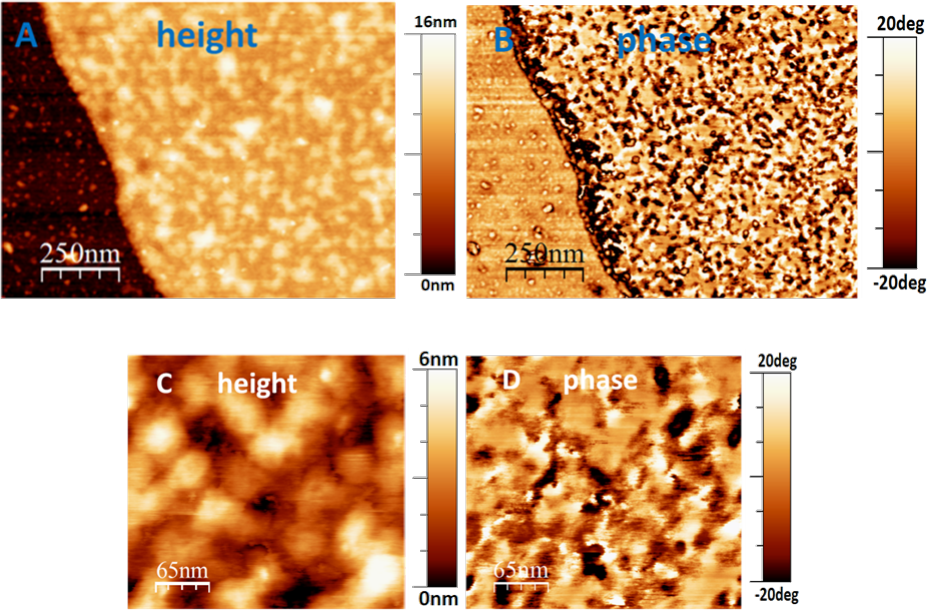
A) AFM height image and B) corresponding phase image of the edge of an annealed CPE45 crystal after grafting **1** for one week. The height image suggests that the crystal surface is completely covered by the semiconducting layer. The phase image indicates that large parts of the layer exhibited elastic behavior similar to the stiffness of the silicon substrate. A comparison between C), the AFM height image, and D), the corresponding phase image of the surface of a CPE45 crystal after grafting **1** for one week, indicated that the dark, i.e., soft and dissipative regions were voids where the semiconducting layer was not completely closed.

After one week of grafting of **1**, the crystal surface was almost completely covered with **1** (Figure 9). Due to side reactions of the coupling procedure [24] and crystallization of **1**, some small grains, which were not chemically attached, were found on the substrate, but only to a smaller extent compared to the amount of material attached to the crystal surface. Most of the perylene layer exhibited a rather stiff (elastic) phase contrast, similar to the solid silicon substrate. We tentatively attribute this highly elastic behavior to the formation of (partially) crystalline perylene islands. According to published values, the energy that perylene molecules gain by crystallization is rather high [25,35], consistent with the poor solubility of molecule **1** and corresponding tendency to crystallize in solution. Thus, it seems plausible that molecules **1,** when being densely packed on the surfaces of CPE45 single crystals, show a tendency to order in a crystalline fashion.

The holes (i.e., the empty spaces in between perylene islands) in the grafted layer appeared dark, i.e., soft and dissipative, because the AFM tip could penetrate these holes, which had a size comparable to the diameter of the apex of the tip. The majority of the islands on the polymer crystal surface had a height between 1 and 3.5 nm. These values compare well with the height of about 1.5 nm for **1** molecules oriented perpendicular to the lamellar surface.

## Discussion

### Monolayers of CPE45 nanocrystals as substrates for organic electronics

While the concept of assembling disk-shaped polymer nanocrystals into large monolayers and functionalizing them with semiconducting molecules in order to create organic electronic devices seemed rather appealing, our experiments have revealed that three major drawbacks impede the realization of the concept.

Firstly, due to the variation of the CPE45 nanocrystal diameters and probably also shapes, the area coverage of the monolayers was limited to about 78%. Compression of the monolayers did not result in a significant improvement of the order or the packing density of the nanocrystal film, which we attribute to random jamming of nanocrystals. We note that the area coverage of the CPE45 nanocrystal films was close to 82.6%, which is the area coverage where jamming of a monolayer of monodisperse, frictionless disks is expected [17]. As CPE45 nanocrystals were neither monodisperse nor frictionless, jamming at a somewhat lower area coverage seems reasonable. This finding may present a general problem when trying to create well-ordered monolayers of platelet-shaped polymer nanocrystals.

Secondly, the tendency of disk-shaped particles to stack was much higher than for spherical particles, probably due to the larger area of contact between disk shaped particles resulting in a larger adhesive van der Waals energy.

**Figure 10:**
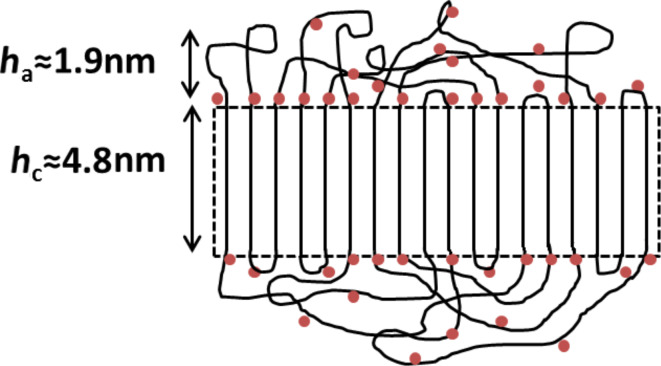
Schematic representation of the morphology of CPE45 nanocrystals, based on results deduced from the height analysis of AFM images. The crystalline core is expected to be surrounded by layers consisting of folds and amorphous segments. In general, the thickness of the resulting amorphous layer depends on crystallization and annealing conditions. The value of *h*_a_ = 1.9 nm represents an average value extracted from Figure 2D.

Thirdly, we found that the carboxyl groups on the surfaces of CPE45 nanocrystals were only partly accessible for chemical reactions. From the analysis of AFM height images of CPE45 nanocrystal films, we found that most nanocrystals were actually considerably thicker than 5 nm. Therefore, we concluded that surfaces of nanocrystals were not covered by carboxyl groups only, but contained also a significant amount of amorphous chain segments connecting sequential carboxyl groups (see Figure 10).

Due to the exclusion of carboxyl groups from the crystal lamella, the thickness of the resulting crystalline core *h*_c_ is about 5 nm. This value has been observed by cryo-TEM images of CPE45 nanocrystals [8]. However, cryo-TEM cannot detect amorphous polyethylene segments, as their density is similar or even lower than that of the surrounding ice [36]. Measuring a characteristic nanocrystal thickness of about 9 nm by AFM thus indicates that the lamellar crystals were covered by amorphous fold layers with thicknesses of about 2 nm.

The presence of an amorphous layer on the surface of CPE45 nanocrystals may account for the low intensity of the emission spectra obtained from CPE45 nanocrystals after grafting **1** molecules to their surface. After nanocrystal formation, a substantial portion of the carboxyl groups were buried under a layer of amorphous chain segments at the fold surface of the nanocrystals. Only a limited number of carboxyl groups within this amorphous layer were accessible for grafting **1** molecules. Hence, at such low surface density of carboxyl groups, the attached dye molecules were widely separated from each other. Therefore, dye molecules could not form aggregates, which can account for the observed emission spectrum (Figure 5A).

While we were able to enhance the density of carboxyl-groups on the fold surfaces of large solution-grown CPE45 single crystals by an appropriate annealing procedure, such a procedure did not yield the same surface enrichment effect for layers of nanocrystals. Due to the high surface to volume ratio of the CPE45 nanocrystals, Ostwald ripening (i.e., fusion of nanocrystals) is expected to take place upon thermal annealing of nanocrystal layers [37–38]. We note that the ratio of lateral surface to volume of a CPE45 nanocrystal is about three orders of magnitude larger than the ratio of lateral surface to volume of a micrometer sized CPE45 single crystal. When the molecules were given enough thermal energy to change their position, molecules which were located close to the edge of the nanocrystals could be redistributed between the nanocrystals. Exchange of polymers between individual nanocrystals occurred until crystalline objects with the least surface have been formed. Under the chosen annealing conditions, this constant exchange of chains most likely caused rather imperfect crystalline domains and partially amorphous fold surfaces with only few carboxyl groups accessible for a chemical reaction.

### Large CPE45 single crystals as substrates for organic electronics

In contrast to the CPE45 nanocrystals, annealing of CPE45 single crystals led to a significant increase of the density of accessible carboxyl groups at the crystal surface, allowing the covalent attachment of a compact layer of semiconducting molecules. As opposed to CPE45 nanocrystals, the driving force for the exchange and reorganization of polymer chains during thermal annealing was low, because the fraction of molecules close to the edge of the crystals was much smaller. Therefore, the number of polymers diffusing in the crystal was extremely small. Thus, during thermal annealing, mainly rearrangements at the level of the fold surface could take place, leading to improved order of the fold surface and a higher density of accessible carboxyl groups, which allowed for a successful reaction with semiconducting molecules.

After grafting **1** for one day to the fold surface of such annealed large single crystals, many islands of semiconducting molecules, and probably many additional non-aggregated **1** molecules, were observed by AFM. However, after longer grafting times (e.g., after three days) islands of **1** molecules formed percolating pathways on the crystal surface. We note that the π–π stacking energy of perylene molecules is rather high [25], consistent with a high melting point and poor solubility of the resulting crystalline aggregates. Hence, crystalline islands of semiconducting **1** molecules are expected to grow preferentially along the π–π stacking direction of the perylene molecules [33–34]. Accordingly, during grafting, incoming molecules were probably guided by the ones already present at the lateral crystal surface. Assuming a high nucleation density for such crystalline islands, even at early stages these growing islands coalesced and merged frequently, leading to the interconnected structures shown in Figure 8.

After a grafting time of one week, almost the entire fold surface of a large CPE45 crystal was covered by domains of aggregated **1** molecules, which were highly elastic and therefore most likely densely packed. The average height of this layer of **1** molecules was between 1 and 3.5 nm. These values cover a thickness range between a single and a double layer of **1** molecules, assuming that all **1** molecules were oriented perpendicular to the lamellar fold surface. The tendency to form double layers may be caused by the asymmetric molecular structure of the perylene monoimide **1**. Thus, chemical functionalization of annealed CPE45 single crystals allowed for the formation of compact layers of semiconducting molecules with an exceptionally low thickness.

## Conclusion

We have assembled self-stabilized polyethylene nanocrystals of CPE45 in monolayers. Due to certain variations in size and shape of these nanocrystals, the arrangement of the nanocrystals did not exhibit long range order but rather a random close packing with a surface coverage of about 78 ± 4%. Carboxyl groups of CPE45 were not easily accessible at the surface of nanocrystals, preventing the attachment of semiconducting molecules. Thermal annealing of the CPE45 nanocrystals did not result in increasing the areal density of semiconducting molecules that could be attached to their fold surface, as annealing of polymer nanocrystals induced Ostwald ripening [37–38] instead of reorganization of the fold surface. Consequently, for the chosen experimental conditions, these nanocrystals did not allow for the attachment of a large number of semiconducting molecules and did not allow the formation of compact semiconducting layer. This hampers the envisioned organic electronics application.

We have therefore grown large lamellar CPE45 crystals. After an appropriate annealing procedure, the CPE45 single crystals allowed for the covalent attachment of molecules at a high areal density, resulting in the formation of a compact layer of semiconducting molecules chemically attached to the crystal surface. We note that the surfaces of the annealed CPE45 single crystals had a remarkably low roughness. Thus, the semiconducting perylene molecules, which were assembled at the crystal surface, probably exhibited a strong overlap of their π-orbitals, which is one of the main prerequisites for efficient charge transport [20]. Finally, we want to emphasize that the perylene derivative **1** used in this study can be replaced by many other semiconducting molecules, which can be covalently attached to the CPE45 crystal surface. Thus, the mono-lamellar crystalline CPE45 surfaces reported here represent a versatile platform for the investigation of the electronic properties of a broad range of semiconducting molecules.

While surfaces of polymer crystals have been modified before, e.g., via harsh oxidization [39–40], such reactions were not precisely controlled and tended to break polymer chains. Thus, our experiments represent the first controlled chemical functionalization of the fold surfaces of polymer single crystals.

## Experimental

### Polymer and nanocrystal synthesis

Details of the synthesis of the CPE45 polymer and the nanocrystals have been published elsewhere [8]. In brief, acyclic diene metathesis (ADMET) polymerization of an carboxylic acid functionalized α,ω-diene monomer was used, which was prepared in a multistep synthetic approach. Nanocrystals were generated by injection of a hot THF solution of CPE45 into a solution of caesiumhydroxide at pH 12 and ultrasonicated for 10 min. The dispersion (initial polymer concentration ca. 1 mg/mL) was filtered through a syringe filter. The dispersions were annealed for five hours at 90 °C and cooled to room temperature over a period of one hour. The resulting nanocrystal dispersions were dialyzed for 30 min against an aqueous solution of potassium hydroxide at pH 11. Experiments with polyethylene surfaces decorated by carboxyl groups have shown that at least pH 11 is needed for complete ionization of the carboxyl groups [41]. As the ionization of carboxyl groups stabilizes the nanocrystals against aggregation, for our experiments we used pH 11 for the dispersion of CPE45 nanocrystals.

### Self-assembled monolayers

The aqueous stock dispersion of nanocrystals with a concentration of *c* < 1 mg/mL was mixed to equal parts with methanol and filtered through a 220 nm pore size Teflon membrane filter in order to remove precipitated nanocrystal aggregates. A micropipette was used to apply the dispersion to the surface of an aqueous solution of potassium hydroxide at pH 11, contained in a Teflon beaker (*h* = 24 mm, *d* = 19 mm). The resulting film was transferred manually via the Langmuir–Schäfer technique onto a silicon wafer cleaned with acetone. Optical microscopy showed that the substrate was covered by about 10% by rafts of self-assembled nanocrystals.

### Compressed monolayers

The aqueous stock nanocrystal dispersion was diluted with methanol in a ratio of 1:1 or 1:3. These diluted dispersions were spread on a Langmuir trough. The inset of the trough was milled from a solid block of Teflon. It was filled with an aqueous solution of potassium hydroxide at pH 12. On basis of the DLVO theory, we estimated that the barrier against aggregation for CPE45 nanocrystals residing at the air–water interface was highest at pH 11. However, it was suggested in the literature that the addition of an electrolyte to the aqueous subphase aids spreading of nanoparticles from methanol on the aqueous subphase. For charged latex particles, it was found that the concentration of the electrolyte should be at least 10 mmol/L [42], corresponding to pH 12 if alkali-hydroxides were used as electrolyte. The aforementioned aqueous/methanolic nanocrystal dispersion was added in a dropwise manner to the water surface. Methanol caused an increase of the surface pressure. After depositing 5–6 drops, we allowed for complete evaporation of methanol. When the surface pressure had decreased by about 0.5 mN/m, additional 5 drops were deposited. In order to be able to record a surface pressure vs area isotherm, we had to deposit a large amount of the rather dilute dispersion. Thus, the deposition and spreading process took about 30 min. After this deposition procedure, a slightly increased surface pressure of Π > 0 mN/m was measured. As the nanocrystals were not compressed and therefore not pushed into the repulsive part of their interaction potential, their presence was not expected to cause a detectable surface pressure. Thus, we attributed the deviation from Π = 0 mN/m to the contribution of methanol in the aqueous subphase. Therefore, we set the observed value as the reference value Π = 0 mN/m. The deposited layer of nanocrystals was either compressed with a constant velocity (about 1 mm/min) or with variable compression velocity. In the variable compression velocity experiment, the films were compressed with high velocity (about 30 mm/min) until the surface pressure started to increase. At this point, the compression mode was changed from constant velocity to constant surface pressure: A threshold-value of Π = 0.5 mN/m was used for switching from fast compression to constant surface pressure. When the pressure dropped below the threshold-value, the control unit compressed the film until the surface pressure had re-increased to Π = 0.5 mN/m. We continued to compress the deposited layer of nanocrystals for ca. 10 h, at which point we reached the limiting area of the Langmuir trough system. At constant surface pressure of Π = 0.5 mN/m, the average compression velocity was about 0.05 mm/min. Similarly, in constant velocity experiments, after compression the films were left to relax for about 12 h, during which an exponential decay of the surface pressure was recorded. (The surface pressure diagrams recorded during the experiments described above can be found in Supporting Information File 1) The films were transferred with the Langmuir–Schäfer technique to a hydrophobic silicon wafer coated with an octadeclytrichlorsilane (OTS) monolayer.

### CPE45 crystallization, annealing and functionalization procedure

In order to grow single crystals, CPE45 was dissolved in tetrahydrofuran (THF). For the initial dissolution, a combination of heating and ultrasonication was required. Upon cooling, the solution became turbid, indicating the onset of crystallization. To re-dissolve the polymer, heating without ultrasonication was sufficient. In order to grow crystals from the homogeneous CPE45 solution, we applied a method based on local supersaturation: A CPE45 solution with a concentration between 0.001 and 0.01 wt % was heated in a tightly sealed vial to a temperature well above the normal pressure boiling point. In the next step, the vial was placed in a heated metal block where it was cooled to the desired crystallization temperature, which was chosen between 45 and 62 °C. The screw cap was removed from the vial, and a silicon substrate was placed inside of the vial. The solution in the open vial was left to evaporate. In order to adjust the evaporation rate, the vial was covered with a cap made from an aluminum foil in which holes were pinched. Depending on the number of pinched holes and the temperature, the evaporation process could take between 10 and 100 h.

For the concentration and temperature range mentioned above, CPE45 is soluble in THF. However, at the three phase contact line formed by substrate, polymer solution and solvent vapor, the concentration was higher. The flux of solvent towards the contact line due to solvent evaporation led to transport of polymer molecules towards the contact line. A concentration gradient was built up, the magnitude of which depended on evaporation rate and the pinning behavior of the contact line. Eventually, the concentration gradient became so steep that close to the contact line the solution became supersaturated. Accordingly, crystals were nucleated. The flux of solvent towards the contact line provided the growing nucleus with the polymer molecules required for growth. When growing large enough, crystals could act as a pinning site for the polymer solution, thereby increasing the time available for the growth of crystals. The areal density and size of CPE45 crystals depended on the pinning behavior of the contact line.

The crystal habit varied from leaf- or lentil-shaped to a lozenge and hexagonal shape, typical habits of polyethylene single crystals [31,43]. Most CPE45 crystals grown by the local supersaturation method were lentil shaped, a shape found for polyethylene single crystals grown at low supercooling [27,31]. As we have grown CPE45 single crystals at temperatures where the polymer is soluble up to a concentration of at least 0.01 mg/mL, we believe that our growth conditions reflect the case of low supercooling. As polyethylene single crystals grown at similar conditions showed the same crystal habit as found for our crystals, we concluded that the observed crystalline objects were CPE45 single crystals.

After growth, single crystals had to be annealed in order to allow carboxyl groups to segregate to the fold surface of the crystals. Annealing at 85 °C allowed for the required rearrangement of polymer chains, which allowed to reorder chain segments at the crystal surface. From AFM images of annealed crystals, we deduced that the thickness of the crystalline object increased by about 2 nm. As an additional consequence of annealing, holes were appearing within the large lamellar crystals, indicative of lamellar thickening [10]. These morphological changes started at the edge of the crystals and proceeded towards the middle (Supporting Information File 1). For most of the crystals examined by AFM after 24 h of annealing, the process of lamellar thickening had led to a Swiss-cheese-like morphology with a uniform thickness of about 10 nm.

The success of chemical attachment of semiconducting molecules was demonstrated with *N*-(4-aminophenyl)perylene-3,4-dicarboximide (**1**) [21]. The synthesis of **1** and preparation of solutions of **1** is described in Supporting Information File 1. Besides the fact that **1** is semiconducting, it is non fluorescent in a dilute solution. However, **1** becomes fluorescent upon reaction with a carboxyl group. Therefore, the covalent attachment of **1** to the crystal surface is conveniently proven by measuring the fluorescence emission. The coupling reaction was carried out in dimethylformamide (DMF) using a standard peptide coupling reagent (1-[bis(dimethylamino)methylene]-1*H*-1,2,3-triazolo[4,5-b]pyridinium-3-oxid hexafluorophosphate) and *N*,*N*-diisopropylethylamine.


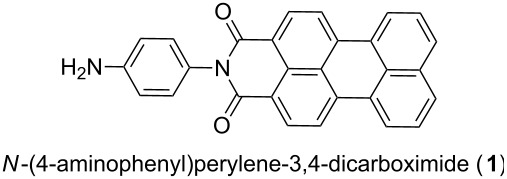


### Analysis of CPE45 single crystals and nanocrystals by AFM

All AFM images of CPE45 nanocrystals and single crystals were recorded in tapping mode on a JPK Nano wizard II. For the analysis of nanocrystal thickness, AFM images of nanocrystal films were plane-flattened. A background surface was subtracted, derived from areas showing the bare substrate. A height histogram as shown in Figure 2D was calculated.

The lateral size of the nanocrystals was extracted by measuring cross sections of closely packing nanocrystals. Phase images as shown in Figure 2 were used. The approximate radius of curvature of the AFM-tip represents the experimental error Δ*x* = 10 nm. A bin size of 5 nm for the cross section was used to determine the distribution of particle diameters. For the AFM images of the nanocrystals, AFM-tips with a nominal radius of curvature of 8 or 10 nm and a resonance frequency around 150 kHz or 320 kHz were used. Both had force constants of the cantilever of about 40 N/m. For the images of the functionalized/non-functionalized CPE45 crystals, sharp AFM tips with resonance frequencies of 40–80 kHz and force constants of 0.12 N/m or 0.24 N/m were used.

### Fluorescence spectroscopy and microscopy

On the stage of an optical microscope (Zeiss Axio Imager A2m), samples were illuminated with a mercury arc discharge lamp (Zeiss HBO 100) through a filter which transmits light in the wavelength range from 450 to 490 nm. Light emission from the crystalline structures was recorded with a CCD camera using a dichroic mirror and a high pass filter, which transmits light with a wavelength above 510 nm. For fluorescence images taken with an objective magnifying by a factor of 100, the detector integrated the emitted light over a duration of 5.1 s. For a 25X objective, this integration time was reduced to 0.5 s. For all registered images, we have used the average of eight subsequently taken images. The background noise was subtracted individually from each color channel of the RGB images. This may have contributed to a small change of spectrum of the emitted light presented in the resulting merged RGB image. We used the same illumination/observation setup of the optical microscope also for photoluminescence (PL) spectroscopy. However, for PL spectroscopy, the detecting CCD camera was replaced by a 400 µm diameter optical fiber which was coupled to an Ocean Optics USB 2000 UV–vis spectrometer. Spectra were recorded with an exposure time of 30 s. Background spectra were recorded on an empty silicon wafer and subtracted from the PL spectra. All spectra were smoothened using Savitzky–Golay filtering. The transmission efficiency of dichroic mirror varied slightly as a function of the wavelength of the transmitted light and thus introduced variations in the measured intensity of the transmitted light. In addition, the sensitivity of the detector decreased significantly for wavelengths above ca. 700 nm. All spectra were therefore corrected for both effects, transmission efficiency of dichroic mirror and sensitivity of the detector.

## Supporting Information

File 1 Supporting information contains (1) surface pressure vs area isotherms of CPE45 nanocrystal films at different pH and surface pressure decay curves of CPE45 nanocrystal films, (2) AFM image and description of lamellar thickening in CPE45 single crystals during thermal annealing, (3) synthesis of the perylene derivative.
