# Supplementary material for: Assembling semiconducting molecules by covalent attachment to a lamellar crystalline polymer substrate
Source: Beilstein J Nanotechnol. 2016 Jun 2;7:784–98. doi: 10.3762/bjnano.7.70 (PMC4902058; doi:10.3762/bjnano.7.70)
Supplement: File 1 — Supporting information contains (1) surface pressure vs area isotherms of CPE45 nanocrystal films at different pH and surface pressure decay curves of CPE45 nanocrystal films, (2) AFM image and description of lamellar thickening in CPE45 single crystals during thermal annealing, (3) synthesis of the perylene derivative. [file Beilstein_J_Nanotechnol-07-784-s001.pdf]

Supporting Information

for

Assembling semiconducting molecules by covalent attachment to a lamellar crystalline polymer substrate

Rainhard Machatschek[1], Patrick Ortmann[2], Renate Reiter[1], Stefan Mecking[2] and Günter Reiter*[1]

Address: [1]Institute of Physics, University of Freiburg, Hermann-Herder-Strasse 3, 79104 Freiburg, Germany and [2]Chair of Chemical Materials Science, Department of Chemistry, University of Konstanz, Universitätsstrasse 10, 78457 Konstanz, Germany

Email: Günter Reiter - guenter.reiter@physik.uni-freiburg.de

* Corresponding author

1. Surface pressure at constant compression rate and constant area pressure relaxation

Figure S1 shows the surface pressure of CPE45 nanocrystal films, which were generated on a Langmuir trough, for an aqueous sub-phase of varying pH, and which were compressed at a constant velocity (ca. 1 mm/min).

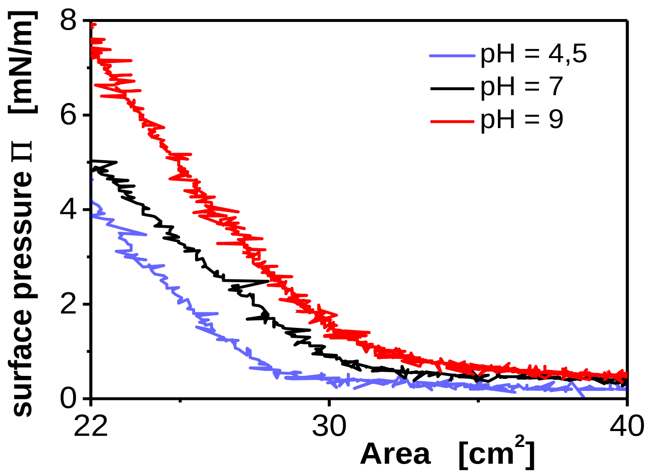

Figure S1: Surface pressure vs. area isotherms of CPE45 nanocrystal films produced at different pH. The scatter of the x-values was caused by the resolution of the electronic control unit. Deviations of the surface pressure from Π=0 at large areas were most likely caused by the methanol used for spreading.

By varying the pH and the ion concentration of the aqueous subphase, we expected to vary the degree of deprotonation of carboxyl groups and therefore to vary the electrostatic repulsion of the nanocrystals. Consequently, we attribute the higher surface pressure observed at higher pH to increased repulsion between nanocrystals.

Furthermore, we investigated the impact of the pH of the subphase and the ion concentration on the decay of the surface pressure relaxation of CPE45 nanocrystal films, at a constant area achieved after compression with constant velocity. Assuming that nanocrystal packing depended on electrostatic repulsion, we anticipated to observe a dependence of the relaxation behavior on subphase electrolyte concentration and pH. All decay curves could be fitted by the sum of a fast and a slow exponential decay, in agreement with a relaxation via

reorganization of particle rafts as a whole (fast relaxation) and particles inside of the rafts [1] (slow relaxation) (Figure S2A). Although these preliminary results do not yet allow for definitive conclusions, the decay curve at pH 12 indicated that the slow relaxations were less significant at high pH, suggesting that the morphology of nanocrystal layers prepared at high pH (and thus high electrostatic repulsion) may differ from the morphology of nanocrystal layers prepared at lower electrostatic repulsion.

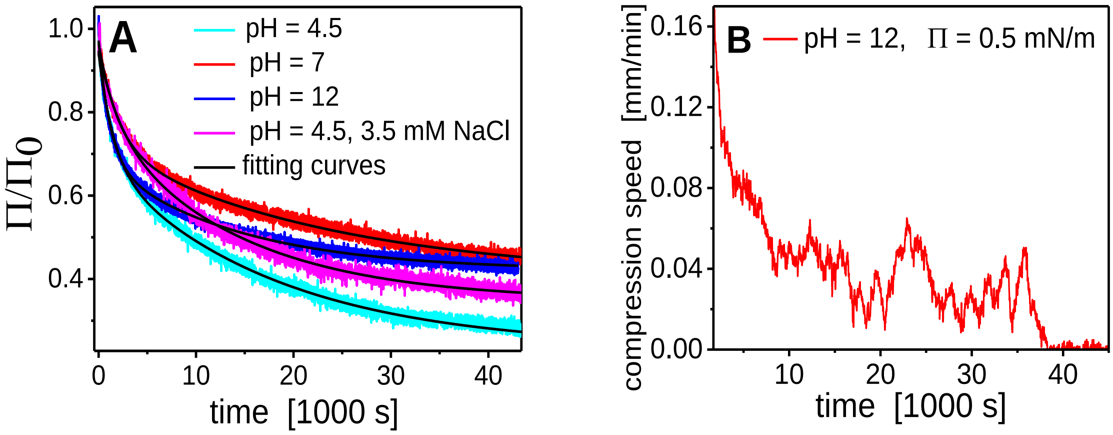

Figure S2: A) Decay of surface pressure of different CPE45 nanocrystal films, which were compressed at constant compression rate. A satisfactory fit of the pressure vs. time curves was achieved with the sum of two exponential decays, having a decay-time on the order of 1000 s and on the order of 10 000 s, respectively. B) Variation of the compression speed for a film, which was compressed at a constant surface pressure of Π = 0.5 mN/m.

The observation of stacks of nanocrystals in transferred films of CPE45 nanocrystal suggested that the films were compressed at a too high surface pressure. Therefore, we have chosen a rather low surface pressure (Π = 0.5 mN/m), which we kept constant by adjusting the compression rate (see Figure S2B). Under such conditions, the area of the film decreased steadily. After 10 hours, no further compression was possible, as we had reached the minimum area of the Langmuir trough.

2. Patterns generated during annealing of large CPE45 single crystals

After annealing at 85 °C for 24 hours, most large-scale crystals possessed a homogeneous lamellar thickness, perforated by some holes induced by lamellar thickening due to mass conservation. However, such crystals required long times for this thickening process and thus allowed to investigate the process of lamellar thickening, e.g., for lentil or leaf shaped CPE45 single crystals.

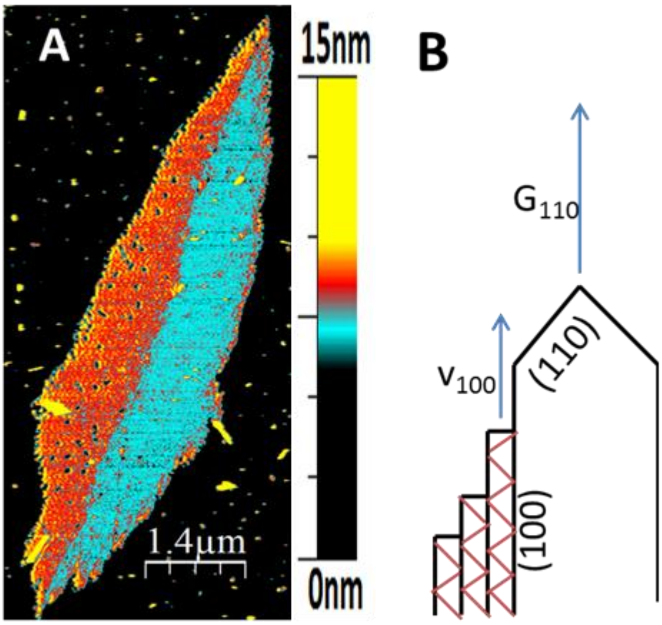

Figure S3: A) AFM height image of an annealed large CPE45 single crystal, which exhibits sectors of slightly different lamellar thickness. B) Schematic representation of the growth tip of a growing polyethylene crystal showing the growth rate of the (110) lattice planes G_{110} and the step propagation rate v_{100} at the (100) planes. The red zigzag line represents the trajectories of the chains in the (100) sectors [1–3].

The AFM height image in Figure S3A shows a crystal, which exhibits sectors of slightly different lamellar thickness, where holes formed almost exclusively in the thicker part of the crystal. For the sector with a smaller lamellar thickness, thickening was much slower and thus one could see that it started at the crystal boundary.

Most likely the rate of lamellar thickening of leaf or lentil shaped polyethylene crystals depends on how the chains are integrated in the crystal. Toda has studied the habits of leaf

or lentil shaped polyethylene crystals grown at low supercooling from the melt [2]. In a very simplified way, one could summarize his interpretation of the lentil shaped habit as a competition between the growth rate perpendicular to the (110) lattice plane G_{110} and the step propagation rate at the (100) lattice plane v_{100} (Figure S3B). When the step propagation at the (100) face cannot keep up with the growth rate in the (110) direction, the crystal grows with a very narrow (110) face and many steps at the (100) surface. The growth perpendicular to the (100) plane requires secondary nucleation of new rows of stems and is therefore the slowest. For a more detailed analysis, we refer the reader to Toda's work [2–4]. As a result, crystal have rounded (100) growth sectors. The red zigzag line in Figure S3B shows the trajectories of the chains in the (100) growth sectors [5]. The beginning of lamellar thickening at the crystal boundary with a propagation towards the center of the crystal suggests that chains, which were attached last, were rearranged first. The narrow (110) growth strip in the middle represents a barrier for the reordering because chains in the (110) sectors are oriented differently than in the (100) sectors, as they are folded parallel to the (110) face [6].

3. Synthesis of the perylene derivative

N-4-aminophenyl-perylene-3,4-dicarboximide (**1**) was synthesized from perylene-3,4,9,10-tetracarboxylic dianhydride and p-phenylenediamine following a procedure given in [7] (Figure S4). The product was a red-to-black powder which was poorly soluble in all tested protic, aprotic, chlorinated and aromatic solvents.

^{1}H NMR (DMSO, 400 MHz, 25 °C): δ (ppm) = 8.98 (s, 2H, Ar**H**), 8.50 (d, 2H, Ar**H**), 8.05 (s, 4H, Ar**H**), 7.83 (s, 2H, Ar**H**), 7.66 (d, 4H, Ar**H**), 3.49 ($\mathbf{H}_2$O), 2.50 (DMSO).

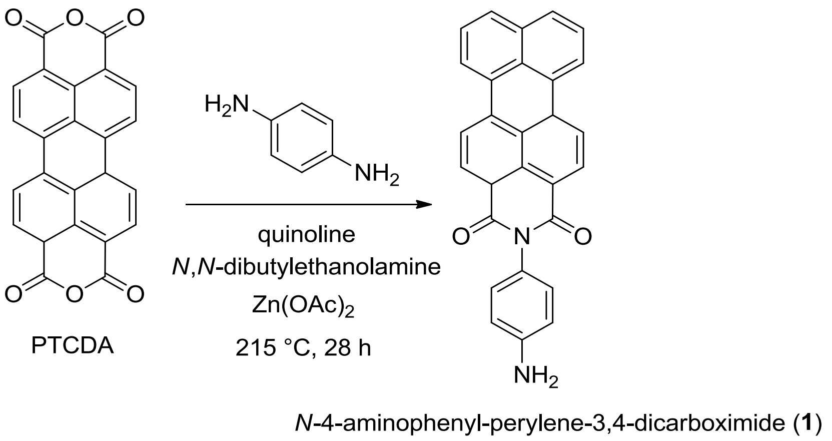

Figure S4: Synthesis of the semiconducting molecules used for functionalization of CPE45.

Best solubility was obtained in dimethylsulfoxide (DMSO), dimethylformamide (DMF) and pyridine. Solutions were prepared by dispersing 0.1 mg/mL of **1** via ultrasonication, followed by heating to the boiling point of the solvent. After filtration, a violet to red solution was obtained. Much of **1** remained in the filter upon filtration, so the concentration was lower than 0.1 mg/mL. Upon storage at room temperature, a red precipitate formed in solutions of **1**. Optical microscopy of the precipitate showed that it consisted, at least partially, of single crystals. Therefore, in order to obtain polymer crystals without any deposits when solutions of **1** were used for functionalization, a second filtration was required prior to addition of solutions of **1** to reaction mixtures.

References

1. Zang, D.; Zhang, Y. *Sci. China Phys. Mech. Astron.*, *54*, 1587–1592.
2. Toda, A. *Colloid Polym Sci* **1992**, *270*, 667–681.
3. Toda, A.; Keller, A. *Colloid Polym Sci* **1993**, *271*, 328–342.
4. Toda, A. *Faraday Disc.* **1993**, *95*, 129.
5. Hocquet, S.; Dosière, M.; Thierry, A.; Lotz, B.; Koch, M.H.J.; Dubreuil, N.; Ivanov, D.A. *Macromolecules* **2003**, *36*, 8376–8384.
6. Ihn, K.J.; Tsuji, M.; Isoda, S.; Kawaguchi, A.; Katayama, K.; Tanaka, Y.; Sato, H. *Macromolecules* **1990**, *23*, 1781–1787.
7. Koenemann, M.; Blaschka, P.; Reichelt, H. Method for producing perylene-3,4-dicarboxylic acid imides. U.S. Pat. Publ. 20080114170 A1, May 15, 2008
